# Legal Requirements for Human-Health Based Appeals of Wind Energy Projects in Ontario

**DOI:** 10.3389/fpubh.2014.00248

**Published:** 2014-12-01

**Authors:** Albert M. Engel

**Affiliations:** ^1^Fogler, Rubinoff LLP, Toronto, ON, Canada

**Keywords:** legal, test, wind turbine, causal link, serious harm, human health

## Abstract

In 2009, the government of the province of Ontario, Canada passed new legislation to promote the development of renewable energy facilities, including wind energy facilities in the province. Throughout the legislative process, concerns were raised with respect to the effect of wind energy facilities on human health. Ultimately, the government established setbacks and sound level limits for wind energy facilities and provided Ontario residents with the right to appeal the approval of a wind energy facility on the ground that engaging in the facility in accordance with its approval will cause serious harm to human health. The first approval of a wind facility under the new legislation was issued in 2010 and since then, Ontario’s Environmental Review Tribunal as well as Ontario’s courts has been considering evidence proffered by appellants seeking revocation of approvals on the basis of serious harm to human health. To date, the evidence has been insufficient to support the revocation of a wind facility approval. This article reviews the legal basis for the dismissal of human-health based appeals.

## Introduction

As of October 1, 2014, allegations of serious harm to human health, resulting from the operation wind farms have been heard and dismissed by the Environmental Review Tribunal (“Tribunal”) in Ontario, Canada a total of 19 times^1^. The reason for this is simple. The evidence put forth by those making the allegations has consistently failed to meet the legal test set out in Ontario’s legislation. The legal test requires those making the allegation to prove, on a balance of probabilities that the wind farm in question will cause serious harm to human health. This paper reviews the health effects that have been alleged, the evidence that has been lead in support of those allegations and the Tribunal’s findings in respect of that evidence.

## Legal Test

In Ontario, since 2009, developers of wind energy projects consisting of wind turbines with a name plate capacity >3 kW are required to obtain a Renewable Energy Approval (“REA”) from Ontario’s Ministry of the Environment and Climate Change (“MOECC”) prior to engaging in the construction and operation of their wind energy project^2^. The issuance of the REA gives the developer provincial approval to proceed with the construction and operation of its project.

Opponents to a wind energy project are provided with the opportunity to appeal the issuance of an REA on two narrow statutory grounds:

(1) “serious harm to human health, and
(2) serious and irreversible harm to plant life, animal life, or the natural environment”^3^.

The Tribunal is furthermore statutorily restricted as to what it may consider when adjudicating over an REA appeal. The statute requires that the Tribunal shall consider only whether engaging in the project *in accordance with the REA, will cause serious harm to human health*^4^.

Finally, the statute places the onus of proving that engaging in the project in accordance with the REA will cause serious harm to human health on the appellant(s)^5^.

If an appellant is able to convince the Tribunal that engaging in a wind energy project in accordance with its REA, will cause serious harm to human health, then the Tribunal gains the jurisdiction to, among other things, revoke the REA. If a project’s REA is revoked, the project can no longer be constructed. This is the ultimate goal of most appellants.

## Constitutional Challenges to EPA Scheme

In addition to alleging serious harm to human health, appellants in *Bovaird, Dixon, Platinum Produce, Wrightman, Drennan, Kroeplin*, and *Fata* have also challenged the constitutional validity of the REA provisions of the EPA (“Charter Challenge”).

In *Bovaird*, the appellants argued that the REA process violated their right to security of the person guaranteed by Section 7 of the *Canadian Charter of Rights and Freedoms* (“*Charter*”).

Section 7 of the *Charter* provides:

7. Everyone has the right to life, liberty and security of the person and the right not to be deprived thereof except in accordance with the principles of fundamental justice^6^.

The Tribunal determined that in order to be successful in their Charter Challenge, appellants needed to show that (a) Section 7 of the *Charter* was engaged; (b) the deprivation of security of the person is serious; and (c) there is proven serious or psychological harm. Ultimately, in *Bovaird*, the Tribunal found that the appellants did not establish serious deprivation of security of the person^7^ or serious psychological or physical harm^8^.

Similar challenges were subsequently brought and dismissed by the Tribunal in the cases mentioned above. Of those cases, *Dixon, Drennan*, and *Kroeplin* have been appealed to Ontario’s Divisional Court on the Charter Challenge issue. Argument is set to occur on November 17–19, 2014 and a decision is expected soon thereafter.

## Tribunal’s Approach to the Legal Test

The Tribunal’s approach to the legal test has consistently been to approach the totality of the evidence put before it according to the entire wording of the test, rather than attempting to artificially subdivide evidence according to the components of the test^9^.

## Categories of Health Effects Alleged

### Direct health effects

Appellants have alleged various direct health effects, resulting from wind turbines. Such direct effects would result in serious physical harm, resulting from audible noise^10^, infrasound^11^, low-frequency noise^12^, shadow flicker^13^, electric and magnetic fields^14^, stray voltage^15^, impacts on gas wells, and sewage lagoons due to corrosion^16^, ice throw^17^, turbine collapse^18^, blade failure^19^, fires^20^, interference with parachutists and pilots^21^, interference with weather radar^22^, and the contamination of drinking water as a result of toxic chemicals and heavy metals leaching from concrete turbine foundations^23^. In all cases, the Tribunal heard extensive evidence that can best be described as raising concerns, but has fallen short of establishing the causal connection required to demonstrate that the various projects, operated in accordance with their REA conditions, will cause serious harm to human health.

### Indirect health effects

Appellants have alleged that serious harm to human health will occur as a result of exposure to wind turbines in the form of indirect health effects such as annoyance, sleep disturbance, headaches, tinnitus, ear pressure, dizziness, vertigo, nausea, visual blurring, tachycardia, irritability, problems with concentration and memory, and panic episodes^24^. While the Tribunal has acknowledged that appellants may meet the legal test by establishing that indirect health effects will result^25^, to date, no appellant has been able to supply the Tribunal with sufficient evidence to do so^26^.

## Lack of Causal Link

In *Erickson*, the Tribunal commented that appellants can attempt to satisfy the legal test even if there is uncertainty about the specific mechanism that causes the alleged health effects^27^. In *APPEC*, the Tribunal expanded on this comment and made it clear that while the specific mechanism of harm need not be established, a causal link must still be established between wind turbines and the alleged health effect:

The Tribunal accepts the findings in *Erickson*, which are unchallenged, that wind turbine noise can cause harm to human health if placed too close to residents. The Tribunal also understands *Erickson* to say that an appellant does not have to establish whether harm is caused by low frequency noise, infrasound, or some other mechanism; however, it is clear from the legal test is s.145.2.1 of the *EPA* that causation must be shown. That is, whether human health is being harmed through direct effects (i.e., audible noise) or indirect effects (i.e., infrasound, low frequency sound, severe annoyance, or by some other mechanism), the appellant must show that the alleged effects are being cause by the project, and by the project when operating in accordance with the REA^28^.

In all cases, the Tribunal has consistently found that appellants have failed to establish such a causal link between wind turbines and direct or indirect human-health effects^29^.

## Categories of Evidence Lead

The cases reveal that appellants have generally lead two types of evidence, which can be described as generic evidence and pre and post-turbine witness evidence.

### Generic evidence

This type of evidence was best described by the Tribunal in *Bovaird* as follows:

Current experience with wind farm projects, both in Ontario, and elsewhere in the world, demonstrates that it is sufficiently predictable that some or all persons living within the vicinity of wind project components (wind turbine(s) being the prominent component) will experience serious health effects. This may be generally described as a generic approach, as it does not seek to establish causation with respect to specific identified individuals. To support their position in this regard the Appellants adduced evidence regarding the incidence of annoyance, which they assert will be caused by wind turbine projects, as well as evidence that environmental noise and annoyance cause stress and sleep disturbance^30^.

The *Erickson* case consisted entirely of this type of evidence. In *Erickson*, the Tribunal concluded that:

The evidence presented by the Appellants, in totality, establishes that there may be an association between exposure to noise from wind turbines and certain indirect health effects, but the evidence is not sufficient to establish a causal connection at the distances and/or noise levels for this Project. The Tribunal finds that the evidence marshalled by the Appellants, such as the Nissenbaum Study and Dr. Aramini’s application of it, is exploratory in nature, not confirmatory. The legal test, however, imposes a standard that requires more than exploratory evidence^31^.

### Pre- and post-turbine witness evidence

Post-turbine witnesses are those who live or have lived in the vicinity of operating wind energy projects. Post-turbine witnesses have testified about serious harm to their health that they believe that they have suffered as a result of living in proximity to wind project components^32^. Pre-turbine witnesses are those who live in the vicinity of the project under appeal and testify about health effects that they believe that they will suffer if the project under appeal is built and operated.

In *APPEC*, the Tribunal heard from 11 post-turbine witnesses who testified about health effects that they believed were caused by living in the vicinity of wind turbines. However, the Tribunal found that it could not rely on their testimony to make the link between their health complaints and wind turbines for the following reasons: (1) expert evidence showing that subjective recall and reporting has been show to be unreliable in scientific studies; (2) factual evidence of unreliable subjective reporting; (3) lack of accompanying noise level measurements; and (4) absence of medical evidence ruling out other causes for the symptoms complained of^33^. The Tribunal also observed that: there is no grouping of symptoms recognized by the medical profession as caused by wind turbines^34^; there are dangers inherent in attempting to draw general conclusions about “wind turbine effects” from anecdotal, personal, and unique experiences^35^; and applying conclusions made from unique personal circumstances at a certain location to projects at other locations is problematic. Furthermore, if a causal connection is established, criteria would need to be identified, which would increase the risk among a certain percentage of the population of having a similar negative health effect^36^.

In *Bovaird*, and several subsequent decisions^37^, the Tribunal commented as follows on why the evidence of post-turbine witnesses was unreliable:

The Tribunal does not question the sincerity of the post-turbine witnesses in giving their evidence. They acknowledge that the identification of their adverse health effects is through their own self-diagnosis. They also acknowledge that they have reached personal conclusions regarding the issue of causation. Several of them assert that they have had to do so, because they maintain that medical professionals either have no knowledge regarding the effects of wind turbines, or are skeptical or dismissive of the possibility that wind turbines can negatively affect human health. Nevertheless, none of the post-turbine witnesses adduced any medical opinion from their health practitioners which confirms that they have experienced symptoms caused by wind turbines. The Tribunal does not question that the post-turbine witnesses have experienced the symptoms they have described. After all, only they can say how they feel. However, in order to arrive at a reliable conclusion respecting causation, personal assessments, which do not consider the full range of potential causes of these symptoms, are incomplete. Furthermore, the exercise of arriving at a diagnosis requires a level of education, training and experience, which none of the post-turbine witnesses possess. In this regard, the Tribunal notes that in Kawartha Dairy, the Tribunal found that confirmation of medical conditions requires the diagnostic skills of a qualified health professional. This conclusion was accepted in APPEC, and the Tribunal accepts that it applies in the circumstances of this case. As discussed below, the Tribunal also does not find that Dr. McMurtry’s opinion about each of the post-turbine witnesses establishes they have experienced adverse health effects caused by wind turbines^38^.

Simply put, the evidence of post-turbine witnesses cannot be extrapolated to conclude that engaging in a project under appeal will cause serious harm to human health because it has not been proven that the health effects complained of were caused by wind turbines^39^. Similarly, a lack of medical evidence has prevented the Tribunal from finding that pre-turbine witnesses with or without particular sensitivities will suffer serious harm as a result of wind turbines^40^. Ultimately, the Tribunal will not assume that a causal link exists between exposure to wind turbines and medical conditions just because post-turbine witnesses sincerely believe there to be one^41^.

## Conclusion

Opponents of wind farms in Ontario have been unsuccessful in appealing REAs on the human-health ground because the Tribunal has consistently found there to be a lack of medical evidence sufficient to support a causal link between wind turbines and reported health effects.

## Conflict of Interest Statement

The author represents developers of wind energy projects in Ontario and has defended the issuance of REAs before the Tribunal in the past.

## Footnotes

^1^*Erickson v. Ontario (Ministry of the Environment)* (2011) OERTD No. 29 (“*Erickson*”); *Middlesex-Lambton Wind Action Group Inc. v. Ontario (Ministry of the Environment)* (2012) OERTD 73 (“*MLWAG 1*”); *Monture v. Ontario (Ministry of the Environment)* (2012) OERTD No.50 (“*Monture 1*”); *Chatham-Kent Wind Action Inc. v. Ontario (Ministry of the Environment)* (2012) OERTD No. 64 (“*CKWA*”); *Monture v. Ontario (Ministry of the Environment)* (2012) OERTD No 69 (“*Monture 2*”); *Haldimand Wind Concerns v. Ontario (Ministry of the Environment)* (2013) OERTD No12 (“*HWC*”); *Alliance to Protect Prince Edward County v. Ontario (Ministry of the Environment)* (2013) OERTD No40 (“*APPEC*”); *MLWAG v. Ontario (Ministry of the Environment)* (2013) OERTD No67 (“*MLWAG 2*”); *Lewis v. Ontario (Ministry of the Environment)* (2013) OERTD No70 (“*Lewis*”); *Bovaird v. Ontario (Ministry of the Environment)* (2013) OERTD No. 87 (“*Bovaird*”); *Dixon v. Ontario (Ministry of the Environment)* (2014) OERTD No5 (“*Dixon*”); *Platinum Produce Company v. Ontario (Ministry of the Environment)* (2014) OERTD No8 (“*Platinum Produce*”); *Drennan v. Ontario (Ministry of the Environment)* (2014) OERTD No10 (“*Drennan*”); *Wrightman v. Ontario (Ministry of the Environment)* (2014) OERTD No11 (“*Wrightman*”); *Bain v. Ontario (Ministry of the Environment)* (2014) OERTD No13 (“*Bain*”); *Moseley v. Ontario (Ministry of the Environment)* (2014) OERTD No23 (“*Moseley*”); *Kroeplin v. Ontario (Ministry of the Environment)* (2014) ERTD No24 (“*Kroeplin*”); *Pitt v. Ontario (Ministry of the Environment)* (2014) OERTD No29 (“*Pitt*”); *Fata v. Ontario (Ministry of the Environment)* (2014) OERTD No42 (“*Fata*”); *Van Den Bosch v. Ontario (Ministry of the Environment)* (2014) OERTD No46 (“*Van Den Bosch*”).

^2^Section 47.2 of the *Environmental Protection Act*, R.S.O. 1990, c.E.19 (“*EPA*”).

^3^Section 142.1(3) *EPA*. The environmental ground in s.142.1(3)(b) of the *EPA* has been included here for completeness, but the balance of the paper will refer only to the human health ground.

^4^Section 145.2.1(2)(a) of the *EPA*.

^5^Section 145.2.1(3) of the *EPA*.

^6^Section 7 of the *Charter*

^7^*Bovaird, supra* note 1 at para. 509.

^8^*Bovaird, supra* note 1 at para. 520.

^9^*Erickson, supra* note 1 at para. 648; *Dixon, supra* note 1 at para. 168.

^10^*Erickson, supra* Note 1; *Monture 2, supra* Note 1; *APPEC, supra* Note 1; *Drennan, supra* Note 1; *Moseley, supra* Note 1; *Kroeplin, supra* Note 1; *Fata, supra* Note 1

^11^*Erickson, supra* Note 1; *Monture 2, supra* Note 1; *APPEC, supra* Note 1; *Bovaird, supra* Note 1; *Dixon, supra* Note 1; *Platinum Produce, supra* Note 1; *Drennan, supra* Note 1; *Wrightman, supra* Note 1; *Bain, supra* Note 1; *Moseley, supra* Note 1; *Kroeplin, supra* Note 1; *Fata, supra* Note 1

^12^*Erickson, supra* Note 1; *Bovaird, supra* Note 1; *Dixon, supra* Note 1; *Platinum Produce, supra* Note 1; *Drennan, supra* Note 1; *Wrightman, supra* Note 1; *Moseley, supra* Note 1; *Kroeplin, supra* Note 1; *Fata, supra* Note 1

^13^*Erickson, supra* Note 1; *Monture 2, supra* Note 1; *Bovaird, supra* Note 1; *Wrightman, supra* Note 1; *Bain, supra* Note 1; *Moseley, supra* Note 1

^14^*Erickson, supra* Note 1; *Monture 1, supra* Note 1; *Monture 2, supra* Note 1; *MLWAG 2, supra* Note 1; *Bovaird, supra* Note 1; *Moseley, supra* Note 1; *Fata, supra* Note 1;

^15^*Monture 1, supra* Note 1; *Monture 2, supra* Note 1; *MLWAG 2, supra* Note 1; *Bovaird, supra* Note 1; *Dixon, supra* Note 1; *Drennan, supra* Note 1; *Wrightman, supra* Note 1; *Moseley, supra* Note 1; *Kroeplin, supra* Note 1.

^16^*Monture 2, supra* Note 1

^17^*Erickson, supra* Note 1; *Platinum Produce, supra* Note 1; *Drennan, supra* Note 1; *Wrightman, supra* Note 1; *Kroeplin, supra* Note 1

^18^*Erickson, supra* Note 1; *Monture 2, supra* Note 1; *APPEC, supra* Note 1; *Drennan, supra* Note 1; *Kroeplin, supra* Note 1

^19^*Erickson, supra* Note 1; *Monture 2, supra* Note 1; *Platinum Produce, supra* Note 1; *Drennan, supra* Note 1; *Wrightman, supra* Note 1; *Kroeplin, supra* Note 1

^20^*Bovaird, supra* Note 1; *Drennan, supra* Note 1; *Wrightman, supra* Note 1; *Moseley, supra* Note 1; *Kroeplin, supra* Note 1; *Fata, supra* Note 1

^21^*Pitt, supra* note 1

^22^*Fata, supra* note 1

^23^*Van Den Bosch, supra* note 1

^24^*Erickson, supra* Note 1; *APPEC, supra* Note 1; *Bovaird, supra* Note 1; *Platinum Produce, supra* Note 1; *Fata, supra* Note 1

^25^*Erickson, supra* Note 1 para 631; *Monture 2, supra* Note 1para 31; *APPEC, supra* Note 1 para 50; paras 171 and 185

^26^*APPEC, supra* Note 1 para. 145, 627; *Bovaird, supra* Note 1 para. 414;

^27^*Erickson, supra* Note para. 819.

^28^*APPEC, supra* Note 1 para. 50.

^29^*APPEC, supra* Note 1 para. 180; *Bovaird, supra* Note 1 para. 377; *Drennan, supra* Note 1, para. 213; *Kroeplin, supra* Note 1 para. 270.

^30^*Bovaird, supra* Note 1 para. 291.

^31^*Erickson, supra* Note 1 para. 828.

^32^*Bovaird, supra* Note 1 para. 292.

^33^*APPEC, supra* Note 1 para. 142.

^34^*APPEC, supra* Note 1 para 143

^35^*APPEC, supra* Note 1 para 175

^36^*APPEC, supra* Note 1 para 175

^37^*Dixon, supra* Note 1 paras. 148, 169, 172–174; *Platinum Produce, supra* Note 1 para. 139; *Drennan, supra* Note 1, para. 225; *Wrightman, supra* Note 1 para. 149.

^38^*Bovaird, supra* Note 1 para. 313.

^39^*APPEC, supra* Note 1 para. 176; *Wrightman, supra* Note 1 para. 151.

^40^*APPEC, supra* Note 1 para. 159–164; *Bovaird, supra* Note 1 paras. 379 and 380; *Drennan, supra* Note 1 para. 197.

^41^*Kroeplin, supra* Note 1 para. 203.

